# Research on Adaptive Grasping with a Prosthetic Hand Based on Perceptual Information on Hardness and Surface Roughness

**DOI:** 10.3390/mi15060675

**Published:** 2024-05-22

**Authors:** Yuxuan Wang, Ye Tian, Zhenyu Li, Haotian She, Zhihong Jiang

**Affiliations:** School of Mechatronical Engineering, Beijing Institute of Technology, Beijing 100081, China; bit_wyx@163.com (Y.W.);

**Keywords:** smart prosthetic hand, information integration, adaptive control, sliding inhibition

## Abstract

In order to solve the problems of methods that use a single form of sensing, the ease of causing deformation damage to the targets with a low hardness during grasping, and the slow sliding inhibition of a prosthetic hand when the grasping target slides, which are problems that exist in most current intelligent prosthetic hands, this study introduces an adaptive control strategy for prosthetic hands based on multi-sensor sensing. Using a force-sensing resistor (FSR) to collect changes in signals generated after contact with a target, a prosthetic hand can classify the target’s hardness level and adaptively provide the desired grasping force so as to reduce the deformation of and damage to the target in the process of grasping. A fiber-optic sensor collects the light reflected by the object to identify its surface roughness, so that the prosthetic hand adaptively adjusts the sliding inhibition method according to the surface roughness information to improve the grasping efficiency. By integrating information on the hardness and surface roughness of the target, an adaptive control strategy for a prosthetic hand is proposed. The experimental results showed that the adaptive control strategy was able to reduce the damage to the target by enabling the prosthetic hand to achieve stable grasping; after grasping the target with an initial force and generating sliding, the efficiency of slippage inhibition was improved, the target could be stably grasped in a shorter time, and the hardness, roughness and weight ranges of targets that could be grasped by the prosthetic hand were enlarged, thus improving the success rate of stable grasping under extreme conditions.

## 1. Introduction

The hand is one of the most unique organs of the human body and plays an important role in daily life. Human fingers are very sensitive, so people can quickly sense the texture and roughness of objects that they touch [1], which allows them to adjust their grasping strategies over time when the grasping environment changes. However, some people suffer partial or total loss of hand function due to illnesses or accidents. Inadequate hand function prevents them from performing some of the basic movements required for daily living, seriously reducing their quality of life. Fortunately, with the progress of microelectronics, mechatronics, sensing technology, and other emerging technologies, various kinds of intelligent prostheses with a shape close to human hands, and the ability to flexibly grasp targets in daily life have been developed by scientific research teams in various countries, thus providing solutions for rebuilding the function of the hands.

In the current research on intelligent prosthetic hands, an urgent research direction is that of improving their ability to perceive the targets to be grasped and the environment. Current tactile sensors can be classified as resistive sensors, photoelectric sensors [2,3], capacitive sensors [4,5], piezoelectric sensors [6,7], piezoresistive sensors [8,9], magnetosensitive sensors [10], etc.; they are rich in variety in terms of their detection principles, and the integration and application of these sensors in prosthetic hands are different [11]. When a piezoresistive tactile sensor is subjected to an external force, internal deformation occurs, which causes the sensor’s resistance to change. This change in the resistance of the sensor can be measured to obtain a pressure measurement. The characteristics of this type of sensor meet the requirements for a small size and light weight for use in a prosthetic hand. Yin et al. succeeded in developing a novel resistive microfluidic shear force sensor [12]. This sensor consisted of liquid metal strain gauges embedded in polydimethylsiloxane. It can be applied to a smart prosthetic hand for the detection of fingertip information, such as shear force and vibration. Optical tactile sensors can respond to changes in certain properties of an object, such as its displacement, speed, and shape, by detecting changes in the intensity of light. Unlike other types of sensors, photoelectric sensors have the advantage of not being subject to electromagnetic interference. Sani et al. proposed a method of detecting the displacement of grasped objects based on optical sensors, which provided a new train of thought for the detection of sliding by prosthetic hands [13]. In addition to the aforementioned sensors that use a single principle, it is also possible to create combined sensors using multiple principles by combining the advantages of different sensors. Such combined sensors are capable of detecting more than one type of external parameter, such as pressure, temperature, light intensity, and other information. In Korea, Kim et al. developed stretchable prosthetic hand skin that integrates arrays of ultrathin, single crystalline silicon nanoribbon into strain, pressure and temperature sensors, thereby greatly enhancing the ability of the prosthetic hand skin to sense the external environment [14].

After making the prosthetic hand sensate, the most important thing is to enable the prosthetic hand to adjust its grasping strategy based on perceptual information to improve its grasping effect. Salisbury et al. have equipped a prosthetic hand with the ability to detect whether an object is sliding or not by installing a sensor made of piezoelectric crystals on the thumb of the prosthetic hand to detect the vibration caused by the sliding of the grasped object [15]. If a sliding signal is detected, the prosthetic hand will further clamp the object until the sliding stops, which can inhibit sliding and improve the stability of the grip. Matulevich et al. reduced the gain of the electromyographic (EMG) signals used for prosthetic hand control by using contact feedback between the prosthetic hand and the object, thereby preventing damage to fragile objects that might be caused by excessive gripping forces [16]. Thomas et al. installed two fabric-based haptic sensors on a prosthetic hand to detect the contact position and thumb tip pressure, which provide feedback on contact position in the form of vibrations, and can adaptively adjust the grip force through haptic information to prevent overgripping and object slippage in the absence of visual feedback [17]. These studies have greatly improved the utility and gripping effect of prosthetic hands, but the current performance of prosthetic hands is still not comparable to that of the human hand. Therefore, prosthetic hands can be optimized according to the biological characteristics of the human hand.

Human movement is divided into volitional movement and reflexive movement. Volitional movement is the result of cognition, while reflexive movement is triggered by perception [18]. The human hand can achieve a good gripping effect when gripping fragile and soft objects, and can quickly inhibit sliding when the object slides. This is because a large number of receptors distributed in the skin of the human hand can provide a wealth of tactile information for humans. Therefore, humans can quickly adjust their grip through reflexive movements based on the perceptual information about the characteristics of the object’s surface and the fluctuation in the load force [19,20].

In order to further improve the sensing abilities of the intelligent prosthetic hand so that it can imitate the human hand’s ability to adjust the grasping strategy according to the characteristics of the objects, the following work was conducted in this study: (1) The finger structure of a single-degree-of-freedom prosthetic hand was optimized, and an FSR and an optical fiber sensor were integrated into it to give it the ability to perceive a target’s hardness and roughness. (2) By constructing scenes of human hands grasping objects with different attributes and collecting grip strength data, the perception and response mechanisms of the human system were analyzed, and an adaptive grip control strategy for prosthetic hands based on a human hand’s grasping strategy was established.

## 2. The Grip Control Strategy of Prosthetic Hands Based on Natural Hand Actions

### 2.1. Introduction to the Prosthetic Hand

The human hand is an extremely complex system that evolved from the pectoral fins of ancient fish. The flexibility of hands has been further enhanced with the evolution of humans, which enables humans to use tools flexibly and makes a significant contribution to the development of human civilization [21,22]. At the same time, the human hand is distributed with a large number of tactile receptors, which can be adjusted according to the sensory information when grasping, thus greatly improving the success rate and stability of grasping. Therefore, the design and optimization of prosthetic hands based on human grasping methods can further improve their grasping.

The most widely used prosthetic hand on the market today is a single-degree-of-freedom prosthetic hand. In general, the dimensions of the fingers and palm are designed with reference to those of a human hand. To ensure that the prosthetic hand is able to grasp objects in a stable manner, its resting position is generally designed to resemble the gesture of a human hand when it is ready to grasp an object. Another type of prosthetic hand is the multi-degree-of-freedom prosthetic hand. This type of prosthesis has more motion, and its shape is more similar to that of a human hand, but its mass and volume are larger than those of a single-degree-of-freedom prosthesis. Since a single-degree-of-freedom hand is simple to control and lightweight, the optimization of this device was carried out in this study in order to facilitate experiments.

**Figure 1:**
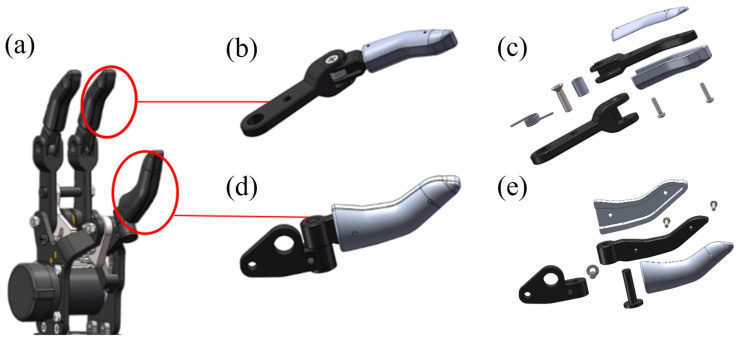
Prosthetic hand structure. (**a**) Overall structure of the prosthetic hand. (**b**) Structure of the index finger. (**c**) Expanded view of the index finger’s structure. (**d**) Structure of the thumb. (**e**) Expanded view of the thumb’s structure.

The main components of a prosthetic hand’s structure are the thumb, index finger, middle finger, motor, and transmission mechanism. The overall structure of a prosthetic hand is shown in Figure 1a. The index and middle fingers are identical in structure and are modular in design. Both the index and middle fingers are made up of proximal, middle, and distal knuckles; the proximal and middle knuckles are designed to be flexible connections, while the middle and distal knuckles are designed to be rigidly integrated. The structure of the index finger is shown in Figure 1b. An expanded view of the index finger is shown in Figure 1c. The index finger mainly contains phalanges, screws, a shaft, torsion springs, and a rubber housing. The thumb of the prosthetic hand consists of the proximal and distal phalanges; the proximal phalanx is connected to the sector gear by a pin and can rotate with the gear. Figure 1d shows the structure of the thumb. Figure 1e shows an expanded view of the structure of the thumb, which mainly comprises phalanges, screws, a shaft, and a rubber housing. When performing a gripping action, the motors cause the index finger to open and close in opposition to the thumb. The middle and index fingers rotate in the same way, and each finger’s distal and middle knuckles move with the proximal knuckles, thereby achieving the gripping function.

The prosthetic hand described herein determines a user’s intent to move by collecting and processing electromyographic signals from the arm. The EMG signals are amplified and filtered and the signals are used by the controller to instruct the hand.

### 2.2. Introduction to the Basic Grip Control Strategy

There are many existing control strategies for prosthetic hands, and stopping when a preset grip force is reached is one of the simpler and more reliable control strategies. In this control strategy, if the control system of a prosthetic hand judges that the user has the intention of grasping, it causes the prosthetic hand to grasp, and when the output value of the sensor of a finger reaches a preset value—meaning that the grasping force has reached a set value—the grasping action stops. In this strategy, it is especially important to preset a suitable value; too high of a preset value can easily cause too much deformation of an object, while too small of a preset value can easily lead to unstable grasping.

**Figure 2:**
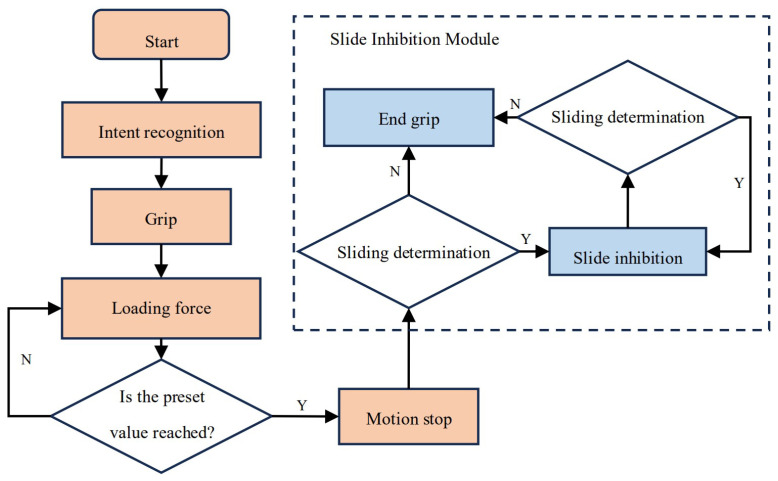
Flowchart of a basic control strategy for a prosthetic hand.

Researchers have conducted many studies on the detection of sliding in prosthetic hands [23,24], so some current prosthetic hands are equipped with the functions of sliding detection and inhibition, but the realization of their functions usually relies on complex sensors and algorithms. When two objects slide relative to each other, there is frictional vibration between them [25], and the vibration intensity is high in the early stage of sliding, so sliding can be sensed by detecting the vibrations [15]. Meanwhile under the influence of the frictional vibration, the contact state between the finger and the object changes, as does the contact force. Therefore, sliding can also be detected according to the change in contact force [26,27,28]. According to this principle, this study used the sudden change in contact force as a simple method of detecting sliding. When the control system detects that the fluctuation in the sensor output signal reaches a threshold that is set in advance (in this study, the threshold was set to 0.1 V in reference to an actual test), this indicates that sliding occurred in the grasping target. The grasping then enters into the second stage, and the control system causes the prosthetic hand to clamp at a preset clamping rate until it reaches a stable state. This sliding inhibition method is relatively simple, but due to the inability to sense the surface information of the grasping target, it is impossible to adjust the clamping rate, resulting in a low efficiency of sliding inhibition. A flowchart of this process is shown in Figure 2. The detection method is applied to the above prosthetic hand control strategy to form the basic control strategy of the prosthetic hand.

### 2.3. Design of a Perception System for the Prosthetic Hand

Prosthetic hands that are practically applied in daily life are currently limited by their cost and reliability, carry fewer types and lower numbers of sensors, and generally adopt simpler control strategies. This kind of prosthetic hand is usually unable to output the appropriate gripping force according to the characteristics of the target, which is easily causes large deformations of the soft target. At the same time, if there is relative sliding between the prosthetic hand and the grasped target in the grasping process, the prosthetic hand cannot adopt the appropriate sliding inhibition method according to the characteristics of the grasped target to improve the sliding inhibition effect. This is mainly because these prosthetic hands cannot acquire enough data to improve their control methods without the appropriate sensors. In order to enhance the sensing abilities of prosthetic hands and improve their grasping effects, this section describes how a prosthetic hand was equipped with new sensors to obtain information on the grasping target and then how the obtained data were processed to improve the control strategy. To address the above issues, we needed to integrate at least two sensors into the prosthetic hand—one for obtaining the hardness information of the target so that the prosthetic hand could differentiate between targets with different hardnesses when grasping and then control the grasping force to reduce the damage to the object, and the other for measuring the surface roughness of the target, so that different sliding inhibition strategies could be adopted according to the different roughness levels of the surface of the target when it slipped, thus realizing fast sliding inhibition.

**Figure 3:**
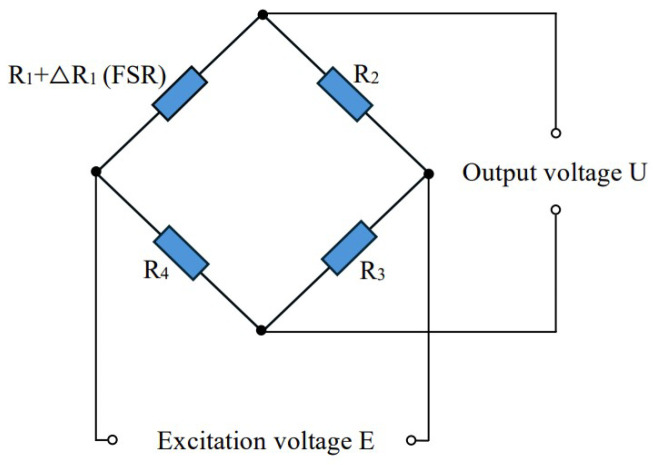
An example of the bridge circuit.

There are many methods of detecting the hardness of objects described in the literature [29,30], but most of them depend on precision instruments and complex algorithms, so they are more suitable for use in laboratories for scientific investigation, and it is difficult to integrate them into a smart prosthetic hand. Considering that the applications of intelligent prosthetic hands have high requirements for their integration and portability, a 10 mm diameter force-sensitive resistor (FSR) was selected for the hardness sensor of the prosthetic hand in this study; it collects the hardness information of the target when in contact with an object and provides an efficient and simple method of distinguishing between the degrees of softness and hardness of the object. During the grasping process, when the prosthetic hand was in contact with the target, the trend in the sensor’s signal varied depending on the hardness of the target. When the prosthetic hand grasps an object with a lower hardness, its finger contact force increases at a slower rate, and the rate of increase in the sensor signal is also slower. When the prosthetic hand’s fingers come into contact with a harder object, the finger contact force rises more quickly, and the sensor signal rises at a higher rate. Thus, the hardness of the gripping target could be represented by the trends in the sensor signal. It should be noted that we use the bridge circuit to convert the resistance change of the FSR into a more obvious voltage change, and the greater the pressure on the sensor, the higher the output voltage. The bridge circuit is shown in Figure 3, where $R_{1}$ is the FSR, $R_{2}$, $R_{3}$ and $R_{4}$ are the balancing resistors, the excitation voltage is E, and the output voltage is U. In order to make the result more intuitive and simplify the follow-up work, we use the voltage change to represent the force change in the following sections.

**Figure 4:**
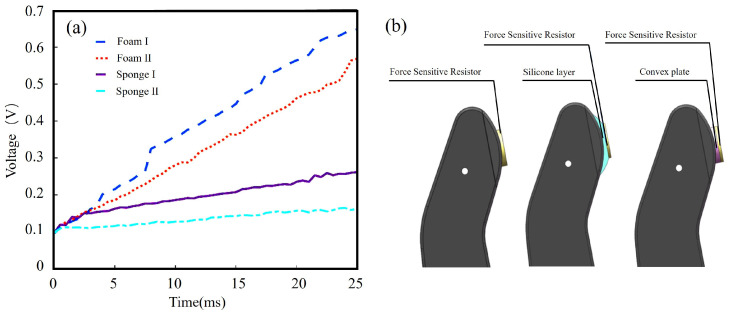
FSR output signals and mounting methods. (**a**) The output signals of the sensor when the prosthetic hand grasps the objects of different hardness. (**b**) Three types of sensor installations.

We verify the validity of this theory by grasping targets of different hardnesses. A curve depicting the changes in the output signal of the sensor after the actual grasping of targets with different hardnesses is shown in Figure 4a. The targets were sorted from hard to soft as follows: Foam I, Foam II, Sponge I, and Sponge II. The x-axis in the figure indicates the contact time, and the y-axis indicates the sensor output signal value. As can be seen in the figure, the output of the FSR presented the following trend: the greater the hardness, the faster the signal rose. At the same time, we found that the difference between signals was more obvious within 0.025 s of contact between the prosthetic hand and the target, and the data points were fewer and less representative when the contact time was less than 0.025 s. When the contact time was more than 0.025 s, the trend of the increase in the signal was similar to that before 0.025 s, which could easily overcomplicate the calculation if included. Therefore, we chose the mean value of the signal of the FSR within 0.025 s of the prosthetic hand made contact with the target to represent the hardness of the grasped target.

As a prosthetic hand performs grasping, it causes different degrees of deformation of its target, leading to the errors in the force being captured by the sensor. Therefore, it was necessary to improve the model of the fingers of the prosthetic hand so that the sensor could obtain more accurate information about the changes in contact force when grasping targets of different hardnesses. We compared the following three methods of prosthetic finger improvement: (1) fixing the FSR directly on a finger; (2) adding a silicone base (which was soft and flexible) to the finger and attaching the FSR to the silicone base; (3) adding a platform that was 10 mm in diameter and 2 mm high under the FSR and integrating that platform into the finger. The platform was hard and not easily deformed. These methods are shown in Figure 4b.

It was found that the second and third improvement schemes were able to improve the measurement of the sensors, but the standard deviation of the signals generated by the sensors after contact with targets of different hardnesses in the third scheme was smaller than those of the other two schemes, so its output was the most stable. Therefore, the third solution was chosen to optimize the finger structure. After determining the mounting scheme, we used the prosthetic hand to grasp objects with different hardnesses and verified that the method could effectively differentiate among the hardnesses of grasping targets.

**Figure 5:**
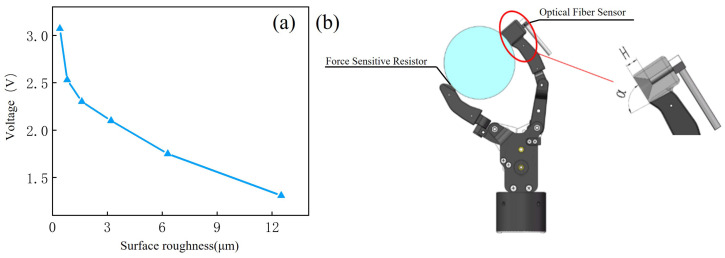
The output and installation method of the optical fiber sensor. (**a**) The corresponding relationship between output voltage and roughness of the optical fiber sensor. (**b**) Prosthetic finger fitted with the fiber-optic sensor.

When a human hand grasps an object, if the object slides, the roughness of the target surface is estimated based on tactile information, and corresponding sliding suppression methods are adopted to achieve stable grasping. This is not possible with a prosthetic hand due to the lack of sensory feedback on roughness. In this section, we propose the use of a laser reflection method to measure the roughness of a target surface; this only required an optical fiber sensor to be placed at a certain distance from the target surface to complete the measurement. In the measurement process, light was first emitted by the fiber optic sensor and was then reflected by the target surface and received by the sensor; the surface roughness of the target was reflected in the amount of light flux received by the sensor. In the case of other factors remaining unchanged, the smoother the surface of the target, the greater the luminous flux of reflected light received. Fiber-optic sensors need to be calibrated before use. We used a roughness measurement instrument and a fiber-optic sensor to measure objects with different surface roughnesses, and the corresponding relationship is shown in Figure 5a. The x-axis represents the results measured with the roughness measurement instrument, and the y-axis represents the output voltage of the fiber-optic sensor. Then, according to the above relationship, the curve fitting is formed to complete the calibration of the sensor.

Mounting the fiber-optic sensor on a prosthetic hand also required us to redesign the prosthetic hand’s fingers. Since the measurement distance and contact angle had an effect on the measurement results of the fiber-optic sensor, in order to find the optimal measurement distance and contact angle, experiments were carried out using fingers with different measurement distances (H = 2 mm, 4 mm, 6 mm) and different tilt angles (α = 90°, 85°, 80°); the fingers are shown in Figure 5b.

After the actual measurement, when the measurement distance was 4 mm and the contact angle was 85°, the measurement results were closest to the results of the roughness measurement instrument, so the above data were applied to the prosthetic hand.

### 2.4. Establishment of Mapping Relationships

The human fingers are covered with dense receptors, so the appropriate initial force can be applied according to a judgment of the hardness of a target at the time of contact; thus, grasping can be achieved without damaging the target. At the same time, human hands can adopt different sliding suppression methods according to the different surface roughnesses of objects, thereby quickly achieving stable grasping. To enable the prosthetic hand to have the ability to perform adaptive grasping like that of the human hand, we needed to analyze the human hand’s grasping mode and establish a corresponding mapping model.

**Figure 6:**
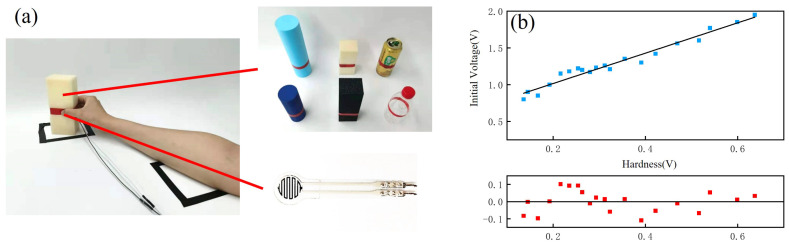
Establishment of hardness–initial force mapping. (**a**) The process of establishing the mapping of hardness to the initial force. (**b**) Mapping of hardness to the initial force.

We applied the same type of FSR to the thumb of a prosthetic hand and to that of a person, and both were used to grasp the same target and quantify its hardness according to the average output of the FSR on the prosthetic hand within 0.025 s of contact with the target; the average of multiple grasping forces of the human hand was used as the expected initial grasping force for grasping the target. In order to obtain the appropriate data for the expected initial grasping force, we collected the grip force of five adults in different scenarios, and their average value was used as the expected standard initial force at this hardness; the volunteers’ ages ranged from 23 to 38. The volunteers were seated in front of a table of the same height for grasping, the arm and target positions were fixed, and the FSR was attached to the tip of the thumb of each volunteer. In order to make the grip conditions of the volunteers’ hands more similar to those of the prosthetic hand, we added a rigid plastic sheet between the finger and the FSR sensor. The grasping position of each grasping target was specified to ensure that there were no large deviations in the gripping position each time, and the experimental environment for acquisition is shown in Figure 6a. By grasping 20 kinds of targets with different hardnesses, 20 sets of data on hardness and initial force were formed.

A linear regression analysis is usually used to explore the quantitative relationship between two variables that affect each other. A linear correlation between the two variables was assumed, which can be expressed by the following equation:

(1) y=kx+b,

where *y* is the predicted value, 20 sets of independent variables $x_{i}$ and dependent variables $y_{i}$ are known, and their corresponding *y* values are predicted from the new *x* values. To establish this mapping, the values of the two parameters *k* and *b* in the model needed to be determined from known data points. This required the development of a metric for determining the optimal values of *k* and *b*. In research, it is common to use a loss function to quantify this metric. The loss function is defined as follows:

(2) $$L=\frac{1}{n}\sum_{i=1}^{n}{\left(y-y_{i}\right)}^{2},$$

where *y* is the predicted value of the model and $y_{i}$ is the true value. The average squared distance between the predicted value and the true value is known as the mean squared error (MSE). Substituting Equation (1) into the loss function and considering *k* and *b* as independent variables of the function *L*, we obtain

(3) $$L\left(k,b\right)=\frac{1}{n}\sum_{i=1}^{n}{\left(kx_{i}+b-y_{i}\right)}^{2}.$$

To find the values of the parameters *k* and *b* that minimize the loss function *L*, *L* can be differentiated from *k* and *b* to obtain

(4) $$\frac{\partial L}{\partial k}=\frac{2}{n}\left(k\sum_{i=1}^{n}{x_{i}}^{2}-\sum_{i=1}^{n}x_{i}\left(y_{i}-b\right)\right),$$

(5) $$\frac{\partial L}{\partial b}=\frac{2}{n}\left(nb-\sum_{i=1}^{n}(y_{i}-kx_{i}\right)).$$

The optimal analytical solutions for the parameters *k* and *b* can be obtained by making the above two equations zero:

(6) $$k=\frac{\sum_{i=1}^{n}y_{i}(x_{i}-\bar{x})}{\sum_{i=1}^{n}x_{i}^{2}-\frac{1}{n}{\left(\sum_{i=1}^{n}x_{i}\right)}^{2}},$$

(7) $$b=\frac{1}{n}\sum_{i=1}^{n}(y_{i}-kx_{i}).$$

The 20 sets of data on hardness and initial force were substituted into the above model to derive the mapping relationship between the initial grip force and target hardness, as shown in Figure 6b, where the x-axis represents the degree of hardness, i.e., the average value of the sensor output after 0.025 s of contact between the finger and the target, and the y-axis represents the average of the sensor output values for all volunteers after they completed a stable grip. The relationship between the two can be expressed in Equation (8). The residuals at the bottom of the figure indicate that data points are available. As can be seen in the figure, the initial gripping force used by the volunteers’ hands in grasping gradually increased as the hardness of the object increased. Since the output voltage of the sensor was positively correlated with the force, the change in voltage will be used to reflect the change in force in the following sections.

**Figure 7:**
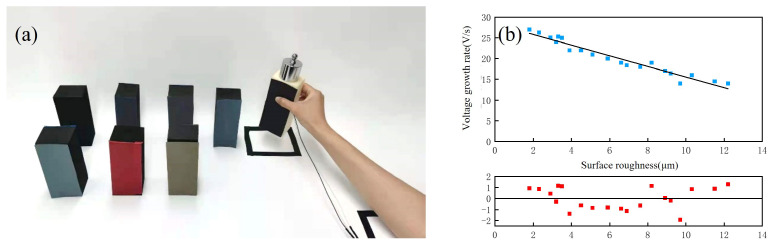
Establishment of surface roughness–growth rate of grip strength mapping. (**a**) Mapping establishment process for surface roughness and the growth rate of grip strength. (**b**) Mapping of surface roughness to the growth rate of grip strength.

(8) y=2.06x+0.6,

When a human hand perceives that a grasped object is sliding, different sliding inhibition strategies are adopted according to the surface roughness of the object. To investigate the relationship between the surface roughness and the sliding inhibition method, five volunteers grasped and inhibited the sliding of 20 objects with different surface roughnesses, and the average rate of increase in the grasping force when inhibiting the sliding of each object was recorded. The same objects were grasped using a prosthetic hand, and their surface roughness was measured and recorded. The acquisition process is shown in Figure 7a. After these steps, two sets of data were formed—one for the surface roughness of the objects and one for the rate of increase in the grip force during sliding inhibition with each roughness. By substituting these two sets of data into the linear regression model, the mapping relationship between the growth rate of the grasping force and the surface roughness of the target was derived, as shown in Figure 7b. The x-axis denotes the surface roughness of the object measured with the fiber-optic sensor, and the y-axis denotes the average rate of increase in the grasping force. The relationship between the two can be expressed in Equation (9). In the figure, it can be seen that as the surface roughness of the grasping target increased, the rate of increase in the force used by the human hand to inhibit its sliding decreased.

(9) y=−1.29x+28.39,

### 2.5. Application of Mapping Relationships to the Control Strategy Based on Perceptual Information

**Figure 8:**
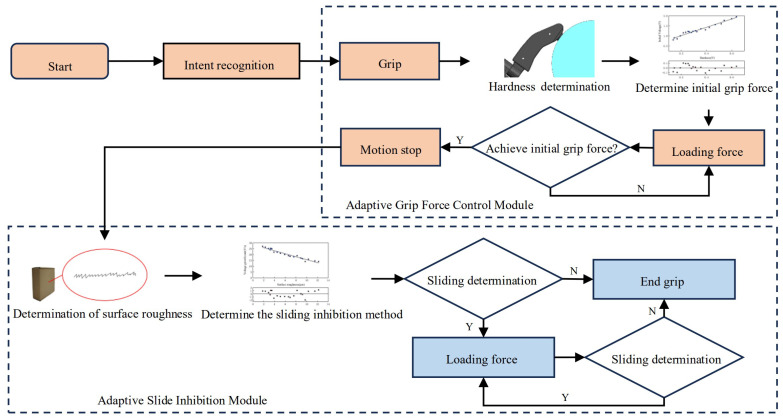
Flowchart of the prosthetic hand control strategy based on information from multiple sensors.

Through the establishment of the above two mapping models, the prosthetic hand was equipped with the ability to use different gripping forces and sliding inhibition methods according to the target’s hardness and surface roughness; a gripping force adaptive control strategy was established for the prosthetic hand, and the flowchart of this is shown in Figure 8.

The prosthetic hand collected myoelectric signals generated by the user through electrodes to judge whether the user had the intention of grasping or not; if the intention to grasp was recognized, the prosthetic hand was directed to grasp the target and then gradually closed until it was in contact with it. When the output value of the FSR was greater than the set haptic threshold, this meant that the fingers of the prosthetic hand were in contact with the grasping target. The control system of the prosthetic hand calculated the average value of the output signal of the FSR within 0.025 s of the prosthetic hand contacting the target and quantified the hardness of the target according to this value. According to the previously established mapping model for the target hardness and desired grip force, the human hand was imitated for the appropriate initial grip force to avoid large deformations of the object. The gripping stopped when the initial gripping force was reached. At this time, the fiber-optic sensor detected the roughness of the target surface. If the control system detected that there was no slippage between the fingers of the prosthetic hand and the target to be grasped, the prosthetic hand maintained the current status quo, and the grasping motion ended. If sliding was detected, the prosthetic hand referred to the trend of the mapping between surface roughness and the growth rate of the grasping force established above, and according to the surface roughness of the grasped target sensed with the fiber-optic sensor, it imitated the human hand in adopting different sliding inhibition methods to achieve the purpose of improving the sliding inhibition efficiency and more quickly achieving stable grasping.

## 3. Prosthetic Hand Grasping Experiments and Results Analysis

The experiments in this section involved the use of a prosthetic hand to grasp targets with different levels of hardness and surface roughness with the control strategy described in this study and the basic prosthetic hand control strategy. By comparing the final grasping force, time taken to inhibit sliding, and grasping success rate with the two strategies, we illustrate the improvement and enhancement of grasping by the prosthetic hand with the grasping force control strategy based on multi-sensor information. Eight able-bodied subjects and two subjects with loss of hand function participated in the experiments.

### 3.1. Grip Force Control Experiments and Results Analysis

**Figure 9:**
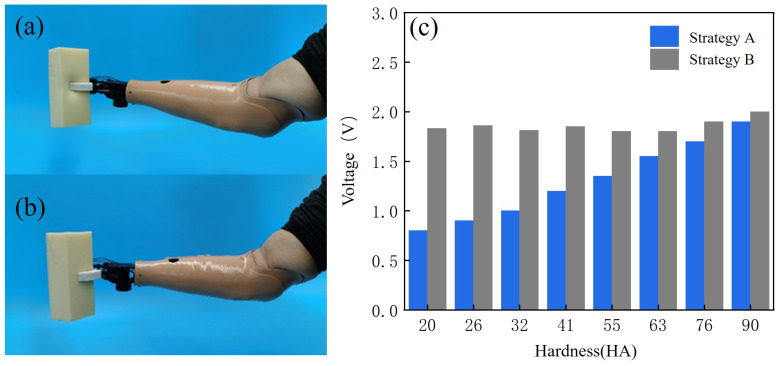
Grip force control experiments. (**a**) Experimental procedure using strategy A. (**b**) Experimental procedure using Strategy B. (**c**) Experimental results.

The 10 subjects wore EMG signal collectors and used the strategy described in this study (Strategy A) to grasp columns with hardnesses of 20 HA, 26 HA, 32 HA, 41 HA, 55 HA, 63 HA, 76 HA, and 90 HA and with sizes of 10 cm × 10 cm × 20 cm, and they did so 10 times each, as shown in Figure 9a. After achieving stable grasping, the grasping force was recorded and averaged; the control group had a prosthetic hand that grasped using the basic control strategy (Strategy B), and the same targets were grasped according to the same method, as shown in Figure 9b. The two groups of experiments did not produce any sliding of the prosthetic hand relative to the target during the grasping process. The final grasping forces of the prosthetic hands under the control of the different grasping force strategies were compared and presented in the form of bar charts, as shown in Figure 9c.

In the comparison graph, it can be seen that, when the prosthetic hand was controlled through Strategy B, the grip force used to grasp targets of different hardnesses was approximated, and the deformations of the targets were greater; when controlled with Strategy A, the grip force of the prosthetic hand increased with the hardness of the target, and there was a difference between the grip force used by the prosthetic hand and that used in the basic strategy when grasping a softer target. The deformations of the target were small, and the grip force used to grasp targets of greater hardnesses was similar to that used in the basic control strategy, which ensured the grasping efficiency. The results of the comparison showed that gripping with the prosthetic hand when using the strategy described in this study resulted in the use of less force to achieve stable gripping according to the hardness of the target, and it caused less deformation of the target.

### 3.2. Sliding Inhibition Experiments and Results Analysis

**Figure 10:**
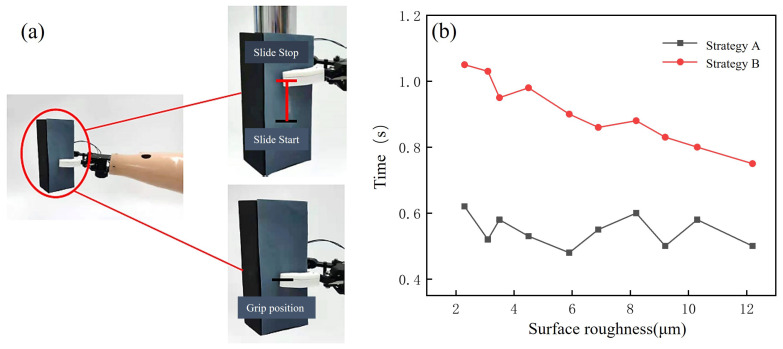
Sliding inhibition experiments. (**a**) Experimental process. (**b**) Experimental results.

Ten subjects were asked to control the prosthetic hand using the control strategy described in this study in order to grasp targets of uniform weight and an approximate hardness with a surface roughness of 2.3–12.2 µm. After achieving a stable grasp, sliding was simulated by adding weights, as shown in Figure 10a. The time taken by the prosthetic hand to achieve a stable grasp by increasing the grip force was recorded. Ten trials with each roughness were carried out by each subject, and the average value was taken. The control group used the prosthetic hand with the basic control strategy; they were made to grasp the same targets, and the time taken to achieve a stable grip after the sliding of the target was recorded. The results of this comparative experiment are presented in the form of a graph in Figure 10b.

In the comparison graph, it can be seen that the time taken by the prosthetic hand to reach steady-state grasping decreased with the increase in the surface roughness of the target when inhibiting the sliding of the grasped target with Strategy B. This was due to the fact that this strategy used a fixed clamping rate, so the time taken to perform sliding inhibition was longer, making it prone to cause grasping failures. When sliding inhibition was performed with Strategy A, the control system of the prosthetic hand adjusted its clamping rate according to the target’s surface roughness, which was sensed in advance with the fiber-optic sensor, and the clamping rate decreased with the increase in the target’s surface roughness. Thus, the clamping rate used in sliding inhibition for smooth targets was higher, and the enhancement of sliding inhibition was more obvious. The results showed that the strategy described in this study was able to improve the sliding inhibition efficiency of the prosthetic hand so that it could more quickly achieve stable grasping.

### 3.3. Gripping Experiments under Extreme Conditions and Results Analysis

In order to research the ranges of hardness, surface roughness, and weight of the targets that could be stably grasped by the prosthetic hand with the two grasping force control strategies, the grasping effectiveness of the prosthetic hand under the two control strategies was compared by controlling the relevant properties of the target that it grasped.

In this experiment, the weight range of the target object that was grasped was between 50 g and 2500 g, the hardness range was between 15 HA and 90 HA, and the surface roughness was between 0.1 µm and 15 µm. We compared the scope of application of the two strategies within the above ranges.

Experiment 1: The subjects used the strategy described in this study (Strategy A) and the basic control strategy (Strategy B) to grasp targets with weights of 400 g, 200 g, 100 g, and 50 g; a hardness of 90 HA; and surface roughnesses of 0.1 µm, 0.8 µm, 1.5 µm, 2.6 µm, 4 µm, and 5.6 µm 10 times.Then, the average grasping success rates of the 10 subjects were calculated and summarized. Cases with a success rate of more than 85% were summarized as the range of applicability of the corresponding strategy.

Experiment 2: The subjects used two strategies to grasp targets with a weight of 100 g, a surface roughness of 15 µm, and hardnesses of 15 HA, 24 HA, 35 HA, 45 HA, 57 HA, and 70 HA 50 times—10 times per group—with a two-minute rest between the groups. The grasping force of a human hand was taken as the standard grasping force. The standard grasping force is a fixed value; however, in the actual grasp, the grasping force used in a successful grasp is not necessarily completely equal to the value of the standard grasping force, as long as there is no large deviation between the two above, we can consider that the grasp is successful, and does not cause large deformation of the target. After the actual grip test, in this experiment, if the voltage corresponding to the final grip force of the prosthetic hand is within ±0.25 V of the voltage corresponding to the standard grip force, it was considered appropriate, and if the prosthetic hand grasped the target stably on this basis, it was regarded as successful. The average grasping success rates of the 10 subjects were calculated and summarized.

Experiment 3: The subjects used two strategies to grasp targets with a surface roughness of 15µm, a hardness of 90 HA, and weights of 400 g, 600 g, 800 g, 1500 g, 2000 g, and 2500 g 50 times each—with 10 times per group—with a two-minute break between groups. The achievement of a final stable grasp was regarded as a successful grasp, and the average grasping success rates of the 10 subjects were calculated and summarized.

In Experiment 1, it was experimentally measured that when the subjects grasped a target with a target weight of 100 g and a surface roughness of 0.1 µm, the grasping success rates under the two strategies tended to be the same and were lower than 85%. When the surface roughness of the target was greater than 0.1µm, the grasping success rate of the prosthetic hand with the two grasping force control strategies was greater than 85% for this type of target.

A comparison of the 10 subjects’ mean grasping success rates in Experiment 2 is shown in Table 1.

The above experimental results show that when controlling the prosthetic hand to grasp the lightweight targets with a rough surface using these two strategies, the success rate of grasping low hardness targets is relatively low, meaning that, in this case, the gap between the grasping force and the standard grasping force was larger, and the prosthetic hand was prone to causing greater deformation in the grasped target during the gripping process. Since the prosthetic hand did not adjust its grip force according to the target’s properties when controlled with the basic strategy and it output a larger grip force when grasping soft targets with a hardness lower than 70 HA, the final grip force was mostly much higher than the standard grip force, and there was a higher probability of serious deformation of the grasped target. It can be considered inappropriate to use this gripping strategy to grip this type of object. Because the prosthetic hand controlled with the strategy described in this study was able to output the corresponding grasping force according to the target’s perceived hardness, it was able to grasp targets with lower hardnesses with a force close to the standard grasping force, but when it grasped targets with very low levels of hardness, the grasping success rate decreased due to the measurement error of the sensor collecting the target hardness information.

A comparison of the 10 subjects’ mean grasping success rates in Experiment 3 is shown in Table 2.

The above experimental results show that, when grasping targets with a high hardness and rough surfaces, the prosthetic hands controlled with the two strategies showed the following tendency: the larger the target’s weight, the lower the grasping success rate. When grasping a target with a weight of 2000 g or more, the sliding suppression method of the prosthetic hand when under the control of the two strategies could not quickly suppress the target’s sliding, resulting in an unsatisfactory grasping success rate; the prosthetic hand under the control of the basic strategy did not adjust the sliding suppression method according to the information of the target’s surface roughness, and the clamping rate of the prosthetic hand on the target was too slow, so it was unable to effectively inhibit the sliding of the target in a timely manner when grasping targets of larger weights. Therefore, when grasping a target with a weight greater than 1500 g, the grasping success rate was low, and it can be considered inappropriate to use this gripping strategy to grip this type of object. The prosthetic hand controlled with the strategy described herein was able to inhibit the sliding of the target by using different sliding suppression methods that imitated a human hand according to the perceived surface roughness, so it was able to reach steady-state grasping more quickly. Therefore, it was able to inhibit the sliding of a target of a greater weight more quickly, thus improving the grasping success rate to a certain extent, but when grasping targets of a very large weight, the clamping rate and grasping force of the prosthetic hand were limited, and the grasping success rate is low, and it can be considered that this strategy is not suitable for this grasping situation.

**Figure 11:**
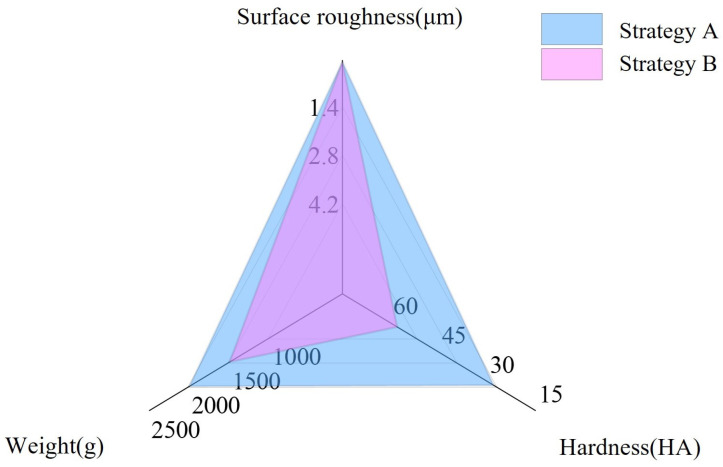
Comparison of the gripping range of the prosthetic hand under the two control strategies.

The cases in which the grasping success rate was more than 85% were summarized as the ranges of applicability of the corresponding strategies, and they are shown in Figure 11. The origin of the coordinate system in the figure was set as follows: 90 HA (hardness), 15 µm (roughness), and 100 g (weight). The results show that the strategies described in this study broadened the ranges of hardness, surface roughness, and weight of targets grasped by this prosthetic hand and improved the success rate of stable grasping in extreme cases.

## 4. Conclusions

In this study, we introduced a control strategy for adjusting the grasping force of a prosthetic hand according to information on a target’s hardness and surface roughness, and we verified the effectiveness of the method through experiments. In order to ensure that the prosthetic hand was able to extract the hardness and surface roughness of the grasping target during the grasping process, we optimized the structure of the prosthetic hand according to the characteristics of the sensors that we used and introduced an information extraction method that improved the ability of the prosthetic hand to perceive the characteristics of the grasping target. After that, we analyzed and modeled the grasping strategy of a human hand, established mapping models between the hardness of the grasping target and the initial force of grasping, as well as the surface roughness of the grasping target and the growth rate of the grip strength, and designed an adaptive grasping control strategy for the prosthetic hand according to these mapping models. Finally, we experimentally compared the grasping effects and scope of applicability of the prosthetic hand when controlled with the control strategy described in this study with those of a basic control strategy.

The experimental results showed that using the grasping strategy described in this study allowed stable grasping to be achieved on the basis of smaller deformations of the grasping target, and a more effective sliding inhibition strategy could be adopted when relative sliding occurred while grasping so as to achieve stable grasping in a shorter time. At the same time, the grip force control strategy described in this study broadened the ranges of hardness, weight, and surface roughness of grasping targets and improved the success rate of the prosthetic hand in achieving stable grasping under the corresponding extreme conditions.

In this study, we installed sensors on the fingers of a prosthetic hand to give it the ability to sense the hardness and surface roughness of an object. However, due to the limitations of the sensor characteristics, the method described in this paper can improve the grasping effect but cannot achieve high-precision control, and at the same time, the sensory information was only used for the control of the prosthetic hand and did not increase the prosthetic user’s perception of the target. In future research, we will focus on how to improve the perception accuracy and control accuracy and reflect them through error analysis, and try to transfer the tactile information from the prosthetic hand to the user so that the user can have a better experience.

## Figures and Tables

**Table 1:** Comparison of the success rates when grasping targets with different hardnesses.

Hardness	Strategy A	Strategy B
15 HA	84.8%	0%
24 HA	90.6%	0%
35 HA	91%	0%
45 HA	91.4%	0%
57 HA	92.2%	32.6%
70 HA	94%	86.4%

**Table 2:** Comparison of the success rates when grasping targets of different weights.

Weight	Strategy A	Strategy B
400 g	98.6%	98.2%
600 g	96.2%	95.4%
800 g	96.4%	94.6%
1500 g	91.6%	85.4%
2000 g	89.4%	78.8%
2500 g	59.2%	46%

## Data Availability

The original contributions presented in the study are included in the article, further inquiries can be directed to the corresponding author.
